# Exploring pancreatic pathology in *Plasmodium falciparum* malaria patients

**DOI:** 10.1038/s41598-018-28797-w

**Published:** 2018-07-11

**Authors:** Supattra Glaharn, Chuchard Punsawad, Stephen A. Ward, Parnpen Viriyavejakul

**Affiliations:** 1 Department of Tropical Pathology, Faculty of Tropical Medicine, Mahidol University, 420/6 Rajvithi Road, Bangkok 10400, Thailand; 2 School of Medicine, Walailak University, 222 Thasala District, Nakhon Si Thammarat 80161, Thailand; 3 Tropical Diseases and Parasitic Infectious Diseases Research Group, Walailak University, 222 Thasala District, Nakhon Si Thammarat 80161, Thailand; 4 Research Centre for Drugs and Diagnostics, Liverpool School of Tropical Medicine, Liverpool L3 5QA, United Kingdom

## Abstract

Hypoglycaemia is an important complication of *Plasmodium falciparum* malaria infection, which can be lethal if not treated. A decrease in blood sugar (BS) level has been correlated with disease severity, parasitaemia and the use of certain antimalarial drugs. This study explored the relationship between pancreatic pathology, including the expressions of insulin and glucagon in the islets of Langerhans, and the BS levels in *P*. *falciparum* malaria patients. Pancreatic tissues from malaria patients were divided into three groups, namely those with BS < 40 mg/dl, BS = 40–120 mg/dl, and BS > 120 mg/dl. In *P*. *falciparum* malaria, pancreatic tissues showed numerous parasitised red blood cells (PRBCs) in the capillaries, oedema, acinar necrosis and the presence of inflammatory cells. The islet size and the expression of insulin were significantly increased in *P*. *falciparum* malaria patients with hypoglycaemia. In addition, insulin expression was positively correlated with islet size and negatively correlated with BS levels. This pioneer study documents an increase in insulin expression and an increase in islet size in hypoglycaemic patients with *P*. *falciparum* malaria. This could contribute to the pathogenesis of hypoglycaemia and provides evidence for the potential need to effectively manage the hypoglycaemia seen in malaria infection.

## Introduction

Malaria caused by *Plasmodium falciparum* is still a widespread parasitic infection causing high morbidity and mortality. The main clinical presentations of severe malaria include cerebral malaria, metabolic acidosis, anaemia, acute kidney injury, pulmonary oedema, renal failure, hypoglycaemia, hypertension and shock^1^. Of these severe manifestations, clinical hypoglycaemia occurs in 8–30% of all severe *P*. *falciparum* malaria cases^2,3^. Children and pregnant women are prone to develop hypoglycaemia in severe malaria possibly linked to lowered immunity. The clinical symptoms of severe hypoglycaemia include hunger, sweating, palpitation, dyspnoea, tachycardia, confusion, anxiety, somnolence, seizure, breathlessness, coma, and conditions may progress to death if not properly treated^1^. The presentation of hypoglycaemia can mimic clinical cerebral malaria but can be differentiated by blood sugar (BS) determination.

Insulin stimulates cells to take up glucose from blood resulting in lower BS levels. On the other hand, glucagon stimulates hepatocytes to release glucose into the blood. Hypoglycaemia in malaria is diagnosed when BS levels are below 2.2 mmol/l or 40 mg/dl^1^. The causes of hypoglycaemia in severe *P*. *falciparum* malaria had been reported to be due to the consumption of glucose by malaria parasites^4^, the effect of antimalarials such as quinine^5^, malarial hepatitis^6^, defects in the gluconeogenesis pathway^7^, the effect of tumor necrosis factor release and the influence of other cytokines produced during malaria infection^8^.

Various studies have described histological changes in the pancreas with infectious diseases. In the study of canine babesia, pancreatic tissues show interstitial and stromal oedema, parenchymal haemorrhages, parenchymal necrosis, fat necrosis and infiltration of a large number of neutrophils^9^. In human pancreatic tissues with leptospirosis, the histopathological changes include oedema, mild inflammatory infiltrate of lymphocytes, haemorrhages, congestion, fat necrosis and calcification^10^. Oedema and pancreatitis were histopathological changes noted in dengue fever^11^. The histopathology of pancreatic tissues with *P*. *falciparum* malaria infection is still incompletely understood and only limited information is available. This study aimed to explore pancreatic pathology in *P*. *falciparum* malaria and to investigate the expressions of insulin and glucagon in the islets of Langerhans in parallel.

## Results

### Summary of clinical data

Pancreatic tissues were obtained from 27 patients who died from *P*. *falciparum* malaria, comprising of 8 cases defined with hypoglycaemia (BS < 40 mg/dl), 9 cases with BS = 40–120 mg/dl, and 10 cases defined with hyperglycaemia (BS > 120 mg/dl). Eight normal pancreas from non-malaria patients were used as controls. No difference in clinical parameters was observed across the groups, including: mean age (years) (26.7 ± 6.0, 21.8 ± 4.2, 33.5 ± 8.1), sex ratio (M:F) (5:3, 6:3, 7:3), number of days of fever (4.3 ± 1.0, 1.3 ± 1.1, 2.9 ± 1.0), haemoglobin level (g/dl) (9.2 ± 0.9, 10.6 ± 1.2, 10.0 ± 1.4), and white blood cell count (×10^3^/μl) (9.2 ± 2.2, 12.1 ± 5.2, 12.2 ± 3.3) (all *P* > 0.05). There was a significant negative correlation between parasitaemia and BS levels (*r*_s_ = −0.610, *P* = 0.046).

### Histopathological changes of pancreatic tissues in *P*. *falciparum* malaria patients

**Figure 1:**
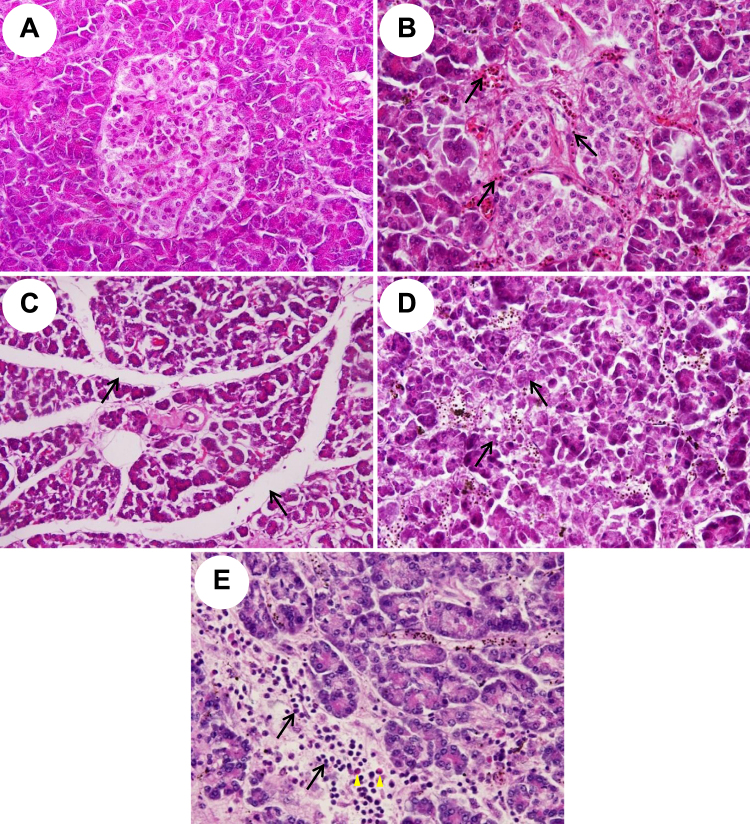
Histopathological changes of pancreas in *P*. *falciparum* malaria patients. Normal pancreas shows unremarkable islets of Langerhans and intact acini cells (**A**, ×400). Pancreatic changes include presence of parasitised red blood cells (PRBCs) within the pancreatic capillaries (**B**- arrows, ×400), oedema in interlobular and intergladular spaces of pancreatic parenchyma (**C**- arrows, ×200), acinar necrosis (**D**- arrows, ×400), and presence of lymphocytes (**E**- arrows), and eosinophils (**E**- arrowheads) in the interlobular spaces (×400).

Pancreatic tissues from *P*. *falciparum* malaria patients usually showed oedema, acinar necrosis and inflammatory reactions, in addition to the presence of parasitised red blood cells (PRBCs). However, there was no difference in the overall total score of histopathological changes between malaria patients and control group (*P* = 0.392). The histopathology of pancreatic tissues from malaria patients, compared to normal pancreatic tissues is illustrated in Fig. 1. Normal histology of the pancreas shows intact islets of Langerhans and acinar cells forming exocrine glands (Fig. 1A). The number of pancreatic islets in malaria patients is similar to the control group (1–2 cells/mm^2^). Numerous PRBCs were frequently seen in the capillaries of pancreas from severe malaria patients (Fig. 1B). Oedema was characterised by widening of the pancreatic interlobular and interglandular spaces (Fig. 1C). There was a significant difference between pancreatic oedema in malaria patients and the control group (*P* = 0.002), especially in malaria patients with BS < 40 mg/dl (*P* = 0.004) and those with BS = 40–120 mg/dl (*P* = 0.004). Necrosis of acinar cells is considered in pale staining cells with the loss of nuclei and visible cellular distortion, causing deformation in pancreatic parenchyma (Fig. 1D). Acinar necrosis was evident in all groups of malaria patients compared to the control group (*P* < 0.001). The presence of inflammatory cells, particularly lymphocytes, neutrophils and occasionally eosinophils in the interlobular septum and interglandular areas were significantly higher in all malaria patients compared to the control group (*P* = 0.011) (Fig. 1E). Fibrosis was not observed in the malarial pancreas. Areas of haemorrhage in pancreatic parenchyma and fat necrosis in peripancreatic tissue were occasionally seen in malaria patients compared to normal pancreas (*P* = 0.196 and *P* = 0.067, respectively). The quantification of total histopathological changes in the pancreatic tissues of malaria patients compared with the control group are shown in Table 1.

**Table 1:** Quantification of pancreatic histopathology in *P*. *falciparum* malaria patients compared to the control group.

Histopathological criteria	Grading score (mean ± SEM)				*P *
	Control	Malaria patients			
		BS < 40 mg/dl	BS = 40–120 mg/dl	BS > 120 mg/dl	
PRBCs	NA	0.67 ± 0.25	0.82 ± 0.34	1.55 ± 0.37	0.224^a^
Oedema	0.44 ± 0.16	1.58 ± 0.23	1.49 ± 0.31	0.82 ± 0.14	0.002^b^
Haemorrhage	0.48 ± 0.23	0.73 ± 0.25	0.98 ± 0.23	1.06 ± 0.22	0.196
Inflammation	0.00 ± 0.00	0.55 ± 0.28	0.71 ± 0.27	0.70 ± 0.18	0.011^b^
Acinar necrosis	0.15 ± 0.05	1.91 ± 0.29	0.95 ± 0.22	1.06 ± 0.24	0.000^b^
Fat necrosis	1.46 ± 0.38	2.20 ± 0.28	1.32 ± 0.33	1.39 ± 0.27	0.067
Fibrosis	0.51 ± 0.16	0.31 ± 0.11	0.33 ± 0.15	1.13 ± 0.23	0.078

^a^Compared between malaria patients.

^b^Significant difference between malaria patients and control group.

### Evaluation of pancreatic islet size

**Figure 2:**
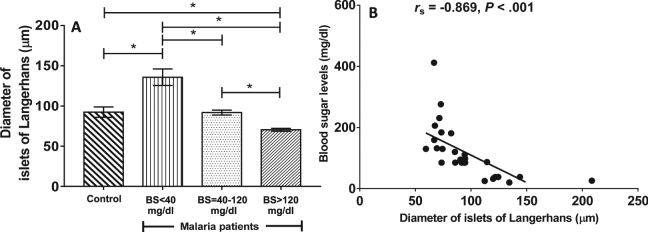
The size of pancreatic islets (µm) of *P*. *falciparum* malaria patients. Data presented as mean ± SEM. *significant difference between groups, *P* < 0.05 (**A**), and negative correlation between islet size, as measured by islet diameter in *P*. *falciparum* malaria patients (n = 27) and blood sugar (BS) levels (**B**).

The comparative size of islets of Langerhans is depicted in Fig. 2A. The diameter of an islet was largest in *P*. *falciparum* malaria with hypoglycaemia (135.80 ± 10.23 µm), compared to *P*. *falciparum* malaria with BS = 40–120 mg/dl (91.89 ± 3.08 µm, *P* = 0.001), *P*. *falciparum* malaria with hyperglycaemia (70.46 ± 1.80 µm, *P* < 0.001), and the control group (95.07 ± 6.82 µm, *P* = 0.004). The calculated area of a pancreatic islet was 15,136.99 ± 2,625.02 µm^2^, 6,702.34 ± 458.21 µm^2^ and 3,922.37 ± 200.76 µm^2^, respectively, as compared to control group (7,012.70 ± 1,088.41 µm^2^). There was a significant negative correlation between the size of pancreatic islets and BS levels (*r*_s_ = −0.869, *P* < 0.001) in malaria patients (Fig. 2B). No significant association was revealed between islet size and other clinical parameters (age, sex, days of fever, parasitaemia, haemoglobin, white blood cell count, albumin and globulin) (all *P* > 0.05).

### Expressions of insulin and glucagon

**Figure 3:**
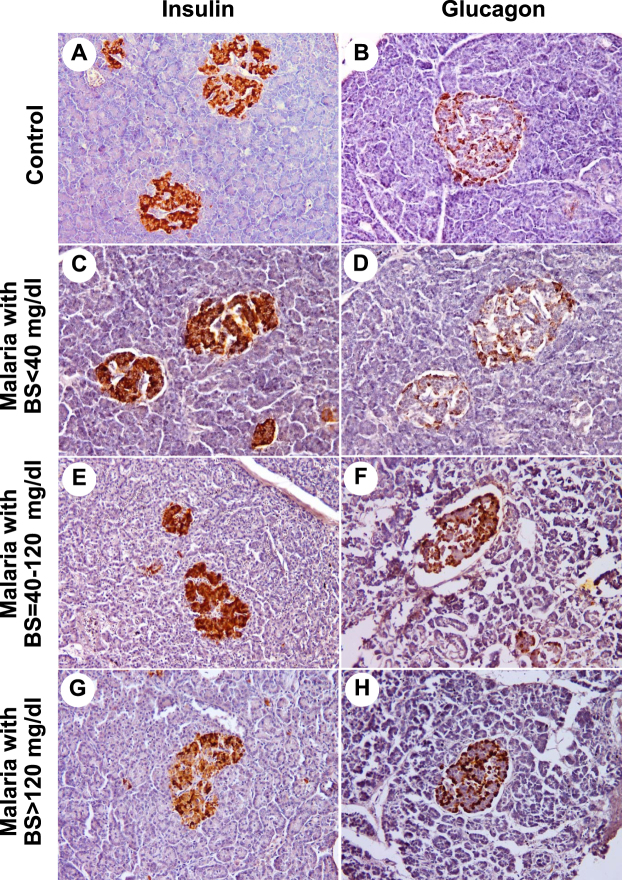
The immunohistochemical staining for insulin and glucagon. Representative sections of human pancreas immunostained for insulin (left panel) and glucagon (right panel) from normal pancreatic tissues (**A** and **B**), pancreatic tissues of *P*. *falciparum* malaria patient with BS < 40 mg/dl (**C** and **D**), pancreatic tissues with BS = 40–120 mg/dl (**E** and **F**) and pancreatic tissues with BS > 120 mg/dl (**G** and **H**). All images are ×200 magnification.

**Figure 4:**
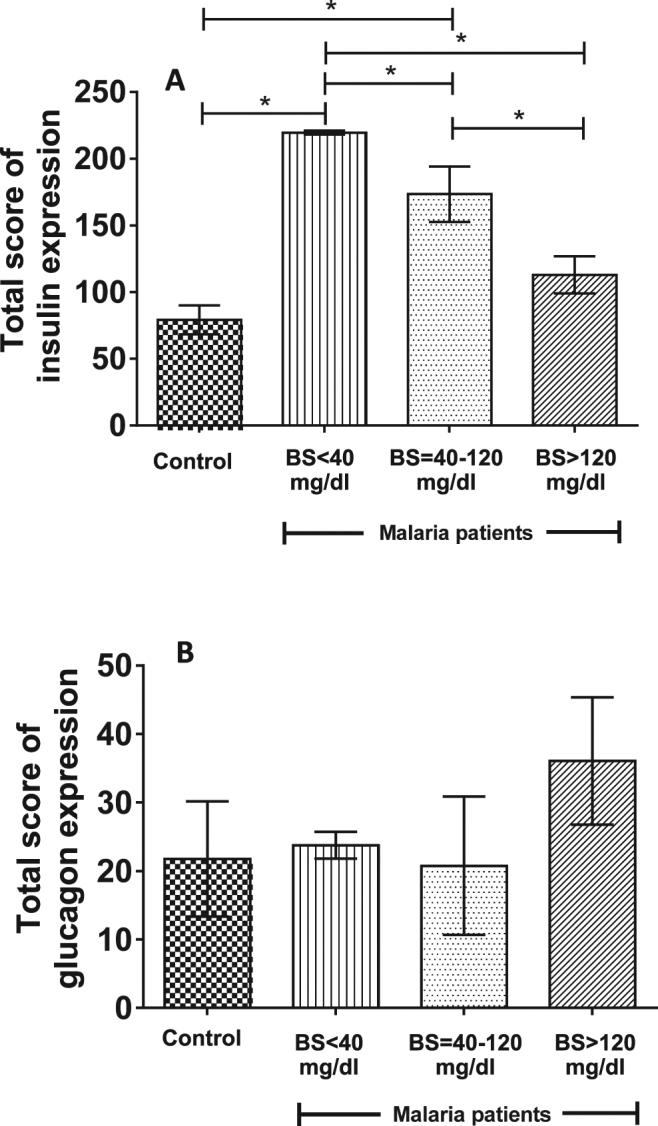
Expressions of insulin (**A**) and glucagon (**B**) in *P*. *falciparum* malaria and control groups. A significant increase in insulin expression was observed in malaria group with BS < 40 mg/dl (*P* = 0.014) and malaria group with BS = 40–120 mg/dl (*P* = 0.011), compared with the control group. No significant difference in glucagon expression was observed in all groups.

**Figure 5:**
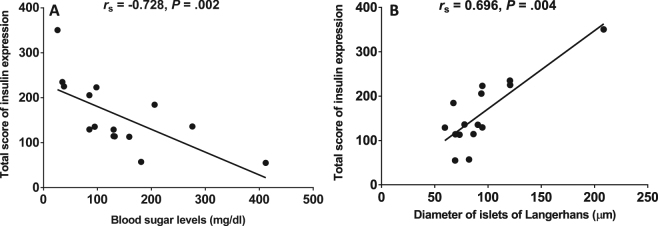
Correlations between BS levels and total score of insulin expressions (**A**) and between islet size and total score of insulin expression in *P*. *falciparum* malaria patients (**B**).

Insulin- and glucagon- expressing cells were detected as brown color in the cytoplasm of the cells in the islets of Langerhans. Generally, insulin expression was demonstrated in the center of the pancreatic islets, whereas glucagon expression was located at the periphery of the islets (control, Fig. 3A,B**)**. In this study, insulin expression was prominent in the group of *P*. *falciparum* malaria patients with hypoglycaemia (Fig. 3C**)**, and those with BS = 40–120 mg/dl (Fig. 3E), compared to the control group (*P* = 0.014 and *P* = 0.011, respectively). A trend towards a reduction in the expression of insulin was revealed in malaria with hyperglycaemia (Fig. 3G). For glucagon expression, weak positive staining was observed in malaria patients with hypoglycaemia (Fig. 3D), whereas moderate positive staining was present in the group of *P*. *falciparum* malaria patients with BS = 40–120 mg/dl (Fig. 3F**)** and the hyperglycaemic group (Fig. 3H). No significant difference in glucagon expression was observed between all malaria patients and the control group (*P* = 0.386). The quantified total expression scores of insulin and glucagon are shown in Fig. 4. There was a significant negative correlation between BS levels and the total insulin expression score (*r*_s_ = −0.728; *P* = 0.002) (Fig. 5A). In addition, insulin expression was directly correlated with islet size (*r*_s_ = 0.696; *P* = 0.004) (Fig. 5B).

## Discussion

Histopathological changes documented in pancreatic tissues of *P*. *falciparum* malaria patients include oedema in the interlobular and interglandular spaces, acinar necrosis and inflammatory reactions. Although these changes were frequently found in *P*. *falciparum* malaria patients with hypoglycaemia (oedema and necrosis), similar histopathological changes were reported in other infectious diseases including dengue infection^11^, leptospirosis^10^ and babesia^9^. Limited studies have been reported on the pathology of pancreatic tissues in severe *P*. *falciparum* malaria. Previous rare autopsy findings showed an enlarged pancreas with minimal fluid in the peritoneal cavity, related clinically to pancreatitis^12^, and necrotic pancreatic tissue^13^. Our findings are similar to reports in animals infected by other plasmodium species. In a study of *P*. *berghei* in mice, pancreatic tissues revealed acute inflammatory reactions of acini, islets cells and interlobular duct^14^. Another study of *Macaca mulatta* monkeys infected with *P*. *knowlesi* showed acinar necrosis and the disappearance of pancreatic islets^15^. In addition, hypoglycaemia was observed in animal models infected with *P*. *knowlesi*^16^. The inflammatory cells such as lymphocytes and neutrophils are crucial in the control of malarial parasite replication, which contribute to the subsequent elimination and resolution of the infection through the process of phagocytosis and/or production of inflammatory mediators^17^. Fibrosis, areas of haemorrhages and fat necrosis in the pancreatic tissues were not features of *P*. *falciparum* malaria patients.

The presence of PRBCs within the capillaries can cause an increase in blood viscosity. In addition, malaria parasites in the red blood cells (RBCs) can cause decrease in RBC membrane deformity. These circulatory disturbances can lead to tissue hypoxia, release of pancreatic acinar enzymes, impaired capillary and venous drainage, which in turn can lead to haemorrhagic pancreatic necrosis^18^. Fibrosis is characterized by the accumulation of extracellular matrix and an increase in fibroblastic reaction. Though fibrosis is not significantly present in *P*. *falciparum* malaria patients, possibly due to the acute nature of malaria infection, a previous study has reported that high BS levels can stimulate the proliferation of pancreatic stellate cells and subsequent increased in fibroblast production^19^.

Whether the increase in islet size is caused by malaria infection per se, or is secondary to antimalarial drugs, needs to be further investigated. Nevertheless, the enlarged pancreatic islets can contribute to hyperinsulinemia and subsequent hypoglycaemia seen in *P*. *falciparum* malaria patients.

The present study used immunohistochemical methods to demonstrate the expressions of insulin and glucagon in terms of distribution and intensity in the pancreatic tissues of *P*. *falciparum* malaria patients. Similar to previous reports, insulin immunopositive cells were found in greater density in the central part of the islets of Langerhans, compared to islet cells expressing glucagon, which are frequently detected around the periphery of pancreatic islets^20^.

The increased expression of insulin in *P*. *falciparum* malaria patients with hypoglycaemia can be secondary to the malaria parasite itself, the use of antimalarial drugs, alteration in the gluconeogenesis pathway, the effects of cytokines during malaria infection and renal injury^6^. Malaria parasites in the circulation need glucose from the host in order to produce energy, mainly via aerobic glycolysis, for their survival^4^. A previous report showed that high parasitaemia is associated with high glucose requirements by malaria parasites, which can lead to clinical hypoglycaemia^21^. Antimalarial drugs, such as quinine and quinidine have an effect on glucose metabolism in malaria patients, causing a decrease in BS level^5^. Most of our malaria patients were treated with quinine. Quinine has been reported to be a more potent stimulant of insulin secretion than quinidine^5^. These drugs act similarly to glucose on potassium permeability of the beta cells membrane, causing calcium influx and subsequent release of insulin to reduce BS levels^5^. We accept that quinine/quinidine have an effect in lowering insulin level and is still used in endemic areas and in places where artesunate is not available. Our data suggests the need for precautious use of quinine/quinidine in these circumstances, and recommend closed monitoring of the BS level of malaria patients. Other antimalarial drugs, such as chloroquine, amodiaquine, mefloquine and halofantrine have no direct effect on insulin secretion^22^. Interestingly, the gluconeogenesis pathway is abnormal in severe *P*. *falciparum* malaria patients due to insufficient galactose substrate, resulting to low BS levels^7^. The effect of cytokines released during *P*. *falciparum* malaria infection, such as tumour necrosis factor (TNF), interleukin (IL)- 1 and 6 can inhibit the activity of phosphoenolpyruvate carboxykinase in the gluconeogenesis pathway and also mediate a reduction in hepatic glycogen content, hence the lower BS levels^23,24^. In addition, IL-1 and 6 have been reported to stimulate islet cell hyperplasia^25^. It can be hypothesised that IL-1 and 6 released during malaria infection can cause islet cell hyperplasia resulting to enlargement of pancreatic islets and subsequent increase in insulin expression. Furthermore, the kidney is an important organ involved in glucose regulation. Occurrence of acute kidney injury in severe *P*. *falciparum* malaria can affect the net glucose release and reabsorption, resulting in hypoglycaemia^26^.

The maintenance of a normal BS level is a critically important process in the metabolism in living organisms. Other parasitic infections have been reported to cause hypoglycaemia, e.g. trypanosomiasis in both human and animals^27^ and Chagas’s disease in a murine model^28^. Although these studies have shown that hypoglycaemia is not a major cause of death, in patients with severe *P*. *falciparum* malaria it is an important clinical manifestation that may well contribute significantly to high mortality rates. This pioneer study documents an increase in insulin expression and an increase in islet size in *P*. *falciparum* malaria patients with hypoglycaemia. The study will be useful for clinicians interested in understanding and managing the process of hypoglycaemia in severe *P*. *falciparum* malaria. The paper sets out recognizable histopathologic and immunologic changes associated with malarial pancreatic tissues.

## Materials and Methods

### Pancreatic tissues

Pancreatic tissues from *P*. *falciparum* malaria patients and control pancreas were obtained from the Department of Tropical pathology, Faculty of Tropical Medicine, Mahidol University, Bangkok, Thailand. For malarial cases, pancreatic tissues were divided into three groups based on the BS levels before death, namely BS < 40 mg/dl, BS between 40–120 mg/dl, and BS > 120 mg/dl. Control pancreatic tissues were obtained from accidental death cases, which showed normal islets of Langerhans, secretory acini, blood vessels, ducts and interstitial area. The study protocol was approved by the Ethics Committee of the Faculty of Tropical Medicine, Mahidol University, Thailand (MUTM 2015–041–01 and MUTM 2015-041-02). Methods were performed in accordance with the relevant guidelines and regulations of the above committee. Written informed consent from closest relatives was obtained for all malaria patients. The Ethics Committee of the Faculty of Tropical Medicine, Mahidol University, Thailand approved the use of control pancreatic tissues obtained from discarded autopsy specimen.

### Histopathological preparation and evaluation

Left-over pancreatic tissues in paraffin blocks were re-embedded and processed using the standard histological protocol. Pancreatic tissues were sectioned at 4 μm thickness for histopathology and immunohistochemistry studies. Histopathological sections of pancreatic tissues were stained with Mayer’s haematoxylin and eosin (H&E). To study the average size of islets of Langerhans, islets were measured and evaluated by randomisation of 40 pancreatic islets under ×100 magnification using the “ImageJ” software program, developed by the National Institutes of Health, Bethesda, Maryland, USA. In addition, the pancreatic tissues were interpreted based on seven histological criteria, namely presence of parasitised red blood cells (PRBCs), oedema, haemorrhage, inflammatory infiltration, acinar necrosis, fat necrosis and fibrosis, under ×200 magnification. The changes were graded on a scale of 0–3. All criteria were assessed in the lobule, interlobular and intergladular areas, except for fat necrosis where the occurrence was at the peripancreatic tissue level. The grading criteria were based on previously described studies^29,30^, with minor modifications (Table 2).

**Table 2:** Histopathological changes and grading criteria for pancreatic tissue evaluation.

Histopathological criteria	Histopathological grading			
	0	1	2	3
Presence of PRBCs	None	<40%/HPF	41–70%/HPF	71–100%/HPF
Oedema	None	interlobular septum	interglandular septum, mild	interglandular septum, severe
Haemorrhage	None	interlobular septum	interglandular septum, mild	interglandular septum, severe
Inflammatory infiltration	None	in 1–2 lobules	in 3–4 lobules	in >4 lobules
Acinar necrosis	None	in 1–2 lobules	in 3–4 lobules	in >4 lobules
Fat necrosis	None	mild	moderate	severe
Fibrosis	None	in 1–2 lobules	in 3–4 lobules	in >4 lobules
Total score: (3) + (3) + (3) + (3) + (3) + (3) + (3) = 21				

### Immunohistochemistry study of insulin and glucagon

The expressions of insulin and glucagon were detected by immunohistochemical staining, using an avidin-biotin peroxidase complex method (Vector Laboratories, Inc., USA). Primary antibodies used were rabbit polyclonal anti-insulin and anti-glucagon (Cell Signaling Technology, Inc., USA). Pancreatic tissues at 4 µm thickness were placed on an adhesive poly-L-lysine slide and heated in a hot oven at 56 °C for 30 minutes. Tissue sections were de-paraffinised and rehydrated in xylene and a decreasing series of graded alcohol, respectively. Then, the pancreatic tissue sections were incubated in 3% hydrogen peroxide diluted in distilled water for 30 minutes at 37 °C in order to block the endogenous peroxidase activity in the tissue. To retrieve the antigen, tissue sections were incubated with 0.1 M sodium citrate buffer at pH 6.0 in a 800 W microwave for 20 minutes. When the slides cooled down to room temperature (RT), the sections were washed in PBS buffer at pH 7.4. To reduce the non-specific background, the sections were incubated with diluted normal goat serum for 30 minutes. Then the pancreatic sections were incubated with a primary antibody (rabbit polyclonal anti-insulin or anti-glucagon (1:200 dilution) in a moisture chamber overnight at 4 °C. The following day, sections of pancreas were washed with PBS buffer at pH 7.4 for three time then incubated with diluted biotinylated goat anti-rabbit Ig G for 30 minutes at RT and treated with avidin-biotin peroxidase complex (ABC) conjugated with horseradish peroxidase (HRP) for 30 minutes. After all sections were washed, the tissue sections were reacted with 0.05% 3,3′ diaminobenzidine (DAB) and 0.01% H_2_O_2_ for 3 minutes, resulting in a brown coloration, which indicated the target antigen. Subsequently, pancreatic sections were counterstained with Mayer’s haematoxylin and mounted with a coverslip for microscopic evaluations.

### Evaluation of immunohistochemical staining

The expressions of insulin and glucagon were evaluated in the cytoplasm of the islet cells in the pancreatic islets. Twenty islets from each pancreatic section were randomly observed at ×400 magnification. The percentages of insulin- and glucagon- expressing cells were determined by counting the number of positive cells over the total number of cells in each islet, multiplied by 100. The intensity of staining with both antibodies was subjectively scored as follows: 0 = no staining, 1 = weakly positive, 2 = moderately positive, and 3 = strongly positive. The total score (TS) was calculated by multiplying the percentage of positive cells (%) and staining intensity (I), according to previous study^31^.

### Statistical analysis

Data were recorded into a computer database and analysed with SPSS software version 18.0. All quantitative data were represented as mean ± standard error of mean (SEM). The test for normality of distribution was calculated using Kolmogorov-Smirnov test. Comparisons of the difference in the size of pancreatic islets between groups were analysed by one way ANOVA. Correlations between variables of histological and immunohistochemical analyses and clinical data were analysed by Spearman’s rank correlation (*r*_s_). Mean differences in the total scores of histopathological and immunohistochemical studies between experimental groups were analysed by Mann-Whitney *U* test. The P-value < 0.05 was considered to be statistically significant.
